# Pivotal Role of Adenosine Neurotransmission in Restless Legs Syndrome

**DOI:** 10.3389/fnins.2017.00722

**Published:** 2018-01-08

**Authors:** Sergi Ferré, César Quiroz, Xavier Guitart, William Rea, Arta Seyedian, Estefanía Moreno, Verònica Casadó-Anguera, Manuel Díaz-Ríos, Vicent Casadó, Stefan Clemens, Richard P. Allen, Christopher J. Earley, Diego García-Borreguero

**Affiliations:** ^1^Integrative Neurobiology Section, National Institute on Drug Abuse, Intramural Research Program, National Institutes of Health, Baltimore, MD, United States; ^2^Center for Biomedical Research in Neurodegenerative Diseases Network and Department of Biochemistry and Molecular Biomedicine, Faculty of Biology, Institute of Biomedicine of the University of Barcelona, University of Barcelona, Barcelona, Spain; ^3^Department of Anatomy and Neurobiology and Institute of Neurobiology, University of Puerto Rico, San Juan, PR, United States; ^4^Department of Physiology, Brody School of Medicine, East Carolina University, Greenville, NC, United States; ^5^Center for Restless Legs Study, Department of Neurology, Johns Hopkins University, Baltimore, MD, United States; ^6^Sleep Research Institute, Madrid, Spain

**Keywords:** Restless Legs Syndrome, periodic leg movements during sleep, hyperarousal, dopamine, glutamate, adenosine, ENT1

## Abstract

The symptomatology of Restless Legs Syndrome (RLS) includes periodic leg movements during sleep (PLMS), dysesthesias, and hyperarousal. Alterations in the dopaminergic system, a presynaptic hyperdopaminergic state, seem to be involved in PLMS, while alterations in glutamatergic neurotransmission, a presynaptic hyperglutamatergic state, seem to be involved in hyperarousal and also PLMS. Brain iron deficiency (BID) is well-recognized as a main initial pathophysiological mechanism of RLS. BID in rodents have provided a pathogenetic model of RLS that recapitulates the biochemical alterations of the dopaminergic system of RLS, although without PLMS-like motor abnormalities. On the other hand, BID in rodents reproduces the circadian sleep architecture of RLS, indicating the model could provide clues for the hyperglutamatergic state in RLS. We recently showed that BID in rodents is associated with changes in adenosinergic transmission, with downregulation of adenosine A_1_ receptors (A1R) as the most sensitive biochemical finding. It was hypothesized that A1R downregulation leads to hypersensitive striatal glutamatergic terminals and facilitation of striatal dopamine release. Hypersensitivity of striatal glutamatergic terminals was demonstrated by an optogenetic-microdialysis approach in the rodent with BID, indicating that it could represent a main pathogenetic factor that leads to PLMS in RLS. In fact, the dopaminergic agonists pramipexole and ropinirole and the α_2_δ ligand gabapentin, used in the initial symptomatic treatment of RLS, completely counteracted optogenetically-induced glutamate release from both normal and BID-induced hypersensitive corticostriatal glutamatergic terminals. It is a main tenet of this essay that, in RLS, a single alteration in the adenosinergic system, downregulation of A1R, disrupts the adenosine-dopamine-glutamate balance uniquely controlled by adenosine and dopamine receptor heteromers in the striatum and also the A1R-mediated inhibitory control of glutamatergic neurotransmission in the cortex and other non-striatal brain areas, which altogether determine both PLMS and hyperarousal. Since A1R agonists would be associated with severe cardiovascular effects, it was hypothesized that inhibitors of nucleoside equilibrative transporters, such as dipyridamole, by increasing the tonic A1R activation mediated by endogenous adenosine, could represent a new alternative therapeutic strategy for RLS. In fact, preliminary clinical data indicate that dipyridamole can significantly improve the symptomatology of RLS.

## BID-induced alterations in the dopaminergic and glutamatergic systems in RLS

Restless Legs Syndrome (RLS) is a very prevalent neurologic disorder. According to the RLS Epidemiology, Symptoms and Treatment (REST) study, 5% of US and European reported experiencing RLS symptoms at least weekly (Allen et al., 2005). Those symptoms include a periodic, rest-induced, mostly nocturnal, movement-responsive urge to move the legs or periodic leg movements during sleep (PLMS) and hyperarousal (Allen et al., 2010; Ferri et al., 2014; Ferré et al., 2015). Thus, RLS patients do not report sleepiness during daytime, even though the total sleep time averages less than 5.5 h (Allen et al., 2010). The deficits of sensorimotor integration that promote PLMS and hyperarousal are interrelated, but there is no obvious cause-effect relationship between the two phenomena. The interrelation can be demonstrated in polysomnographic studies, which allows measuring the relation between the onset and offset of the arousal events and the concomitant onset and offset of PLMS. These studies have shown that, although it is generally believed that PLMS cause the arousal episodes, these precede the onset of PLMS in more than 40% of cases (Ferré et al., 2015). However, their tight temporal relationship suggests that both events are dependent on a common additional mechanism.

Altered dopaminergic function seems to play an important role in PLMS, which is empirically supported by the significant therapeutic response to L-dopa and dopamine receptor agonists, such as pramipexole and ropinirole (Earley et al., 2014). And it is generally believed that because these drugs have a preferential affinity for dopamine D_3_ vs. dopamine D_2_ receptors (D3R and D2R, respectively), that D3R constitute a main target responsible for their therapeutic effects (Varga et al., 2009; Manconi et al., 2011). Nevertheless, there is also evidence of biochemical alterations in the dopaminergic system. The dopaminergic profile in RLS includes abnormally high levels of the dopamine metabolite 3-ortho-methyldopa (3-OMD) in the CSF (Allen et al., 2009), a decrease in the density of striatal D2R and a pronounced increase in tyrosine hydroxylase activity in the striatum and substantia nigra (Connor et al., 2009). This would be mostly compatible with a *presynaptic hyperdopaminergic state*, with downregulation of D2R being probably an adaptation secondary to an increased basal dopaminergic tone (Earley et al., 2014). The presence of a hyperdopaminergic state in the basal ganglia obviously posits the question about the mechanism involved in the therapeutic effect of dopamine receptor agonists.

On the other hand, glutamatergic mechanisms probably play an important role in the RLS hyperarousal component. A magnetic resonance spectroscopy imaging study in subjects with RLS showed a significant increase in the thalamic concentration of glutamate (measured by the proxy variable Glx, which represents glutamate plus glutamine), which correlated with the time spent awake during the sleep period (Allen et al., 2013b). These findings therefore suggest a *presynaptic hyperglutamatergic state* in RLS that could underlie the hyperarousal of RLS. In fact, glutamatergic mechanisms play a central role in the therapeutic effects of α_2_δ ligands, such as gabapentin and pregabalin, which are the main therapeutic alternative to dopaminergic ligands for initial treatment of RLS (Garcia-Borreguero et al., 2013). Thus, α_2_δ ligands bind to an auxiliary regulatory protein (α_2_δ) of voltage-dependent calcium channels that preferentially modulate neurotransmitter release from glutamatergic terminals (Dooley et al., 2007). The α_2_δ ligands are most effective for the sleep disturbances in RLS, but, although less effective than dopaminergic agonists, they are also effective for PLMS, (Garcia-Borreguero et al., 2014). In summary, RLS pathophysiology seems to depend on alterations in two different, but *somehow interrelated*, neurotransmitter systems, dopamine and glutamate. The dopaminergic system is mostly related to the disturbance in sensorimotor integration with the emergence of PLMS and glutamate seems to be involved with both PLMS and hyperarousal.

Brain iron deficiency (BID) is recognized as a main initial pathophysiological mechanism in the development of RLS (Earley et al., 2014). The association between iron deficiency and RLS was originally described by Nordlander (1953). Further studies showed a high prevalence of RLS symptoms in conditions with compromise of iron availability (Allen and Earley, 2007). The prevalence of RLS in a population of patients with iron-deficient anemia was reported to be as high as 31.5% (Allen et al., 2013a), about six times higher than the prevalence for RLS in the general population (Allen et al., 2005). Nevertheless, most patients with RLS do not have systemic iron deficiency. Although, as already proposed by Nordlander, RLS patients present a specific iron insufficient state in the brain. Thus, all studies of CNS iron have consistently shown BID in RLS (reviewed in Earley et al., 2014). This *brain-specific deficit in iron* seems to be related to a dysregulation of iron transportation by the blood-brain barrier. Thus, postmortem studies suggest alterations in the expression or function of iron management proteins in the choroid plexus and brain microvasculature (Connor et al., 2011). It would therefore be appropriate to address RLS as a brain iron dyshomeostasis, a functional disorder of iron acquisition by the brain (Connor et al., 2017). Significantly, there is clinical and experimental evidence for a connection between BID and the alterations in the dopaminergic system in RLS. Autopsy analysis have revealed that the immunostaining for iron management proteins is altered in the substantia nigra of RLS brains and the profile of proteins responsible for iron management in the neuromelanin cells of the substantia nigra indicate iron deficiency (Connor et al., 2004). Furthermore, there is significant literature from animal research that indicates a close relationship between brain iron status and the dopaminergic system (for review, see Earley et al., 2014).

In rodents, BID (including in the ventral midbrain) can be consistently induced by providing a severe iron-deficient diet during the post-weaning period. Even though it does not show motor alterations that would imitate PLMS, the post-weaning, diet-induced BID rodent represents a well-accepted pathophysiological model of RLS (Connor et al., 2009; Earley et al., 2014; Unger et al., 2014). In fact, it provides a biological model for the understanding of the connection of the iron and dopamine alterations in RLS, since it reproduces the main alterations in dopaminergic transmission observed in RLS patients. Those include an increase in striatal extracellular concentrations of dopamine, a reduction in the density of striatal D2R and an increased TH activity in the ventral midbrain (Connor et al., 2009; Unger et al., 2014). Although it does not show motor abnormalities, the BID rodent does reproduce the circadian sleep architecture of RLS, showing an increase in wakefulness at the end of the awake period, which corresponds to the circadian time point where RLS symptoms are associated with maximal disruption of sleep (Dean et al., 2006). This implies that this model could also provide clues for the mechanisms involved in the hyperglutamatergic state of RLS or for alterations in other neurotransmitter systems that could underlie the changes in both the glutamatergic and the dopaminergic systems. A possible candidate is adenosine and its two main receptor subtypes in the brain, A_1_ and A_2A_ receptors (A1R and A2AR). Thus, well-known main functions of adenosine are: first, to exert a brake in the function of the ascending dopaminergic system by presynaptic mechanisms and by postsynaptic mechanisms mediated by receptor complexes of specific adenosine and dopamine receptor subtypes, the A1R-dopamine D_1_ receptor (D1R) and A2AR-D2R heteromers (Ferré et al., 1997, 2016; Ginés et al., 2000; Hillion et al., 2002; Canals et al., 2003); second, to act as a universal A1R-mediated presynaptic inhibitor of glutamatergic transmission (Wu and Saggau, 1997; Dunwiddie and Masino, 2001; Cunha, 2016); third, to act as a mediator of sleepiness induced by prolonged wakefulness, when adenosine accumulates in the extracellular space and acts mostly on A1R localized in basal forebrain, cortex, and hypothalamus (McCarley, 2007; Ferré, 2010). As here reviewed, adenosine neurotransmission can provide the link between dopamine and glutamate mechanisms in RLS and a hypoadenosinergic state can explain the hyperdopaminergic and hyperglutamatergic state of RLS.

## BID-induced alterations in the adenosinergic system

In view of the established functional and molecular interactions between striatal D2R and A2AR (see below), we first investigated possible alterations in the density or function of striatal A2AR associated with BID in rodents. In fact, in three separate studies we found a consistent upregulation of striatal A2AR in rats and rodents with severe BID, which was behaviorally associated with a higher efficacy of A2AR antagonists to produce locomotor activation (Gulyani et al., 2009; Quiroz et al., 2010, 2016a). The A2AR upregulation could also be reproduced in a mammalian cell line upon exposure to an iron chelator (Gulyani et al., 2009). But, in our most recent study, by analyzing receptor density by Western Blot and by radioligand binding assays, we could also demonstrate a pronounced downregulation of A1R both in the striatum and in the cortex, together with the expected downregulation of striatal D2R (Quiroz et al., 2016a). When administering a less severe iron-deficient diet, still associated with BID (as demonstrated by a significant upregulation of transferrin receptor), the same degree of downregulation of A1R and D2R could be observed, but not the A2AR upregulation (Quiroz et al., 2016a). *These results indicate that downregulation of A1R might constitute a more significant clinical correlate of BID in RLS, while changes on A2AR density would be only observed with severe BID*.

**Figure 1:**
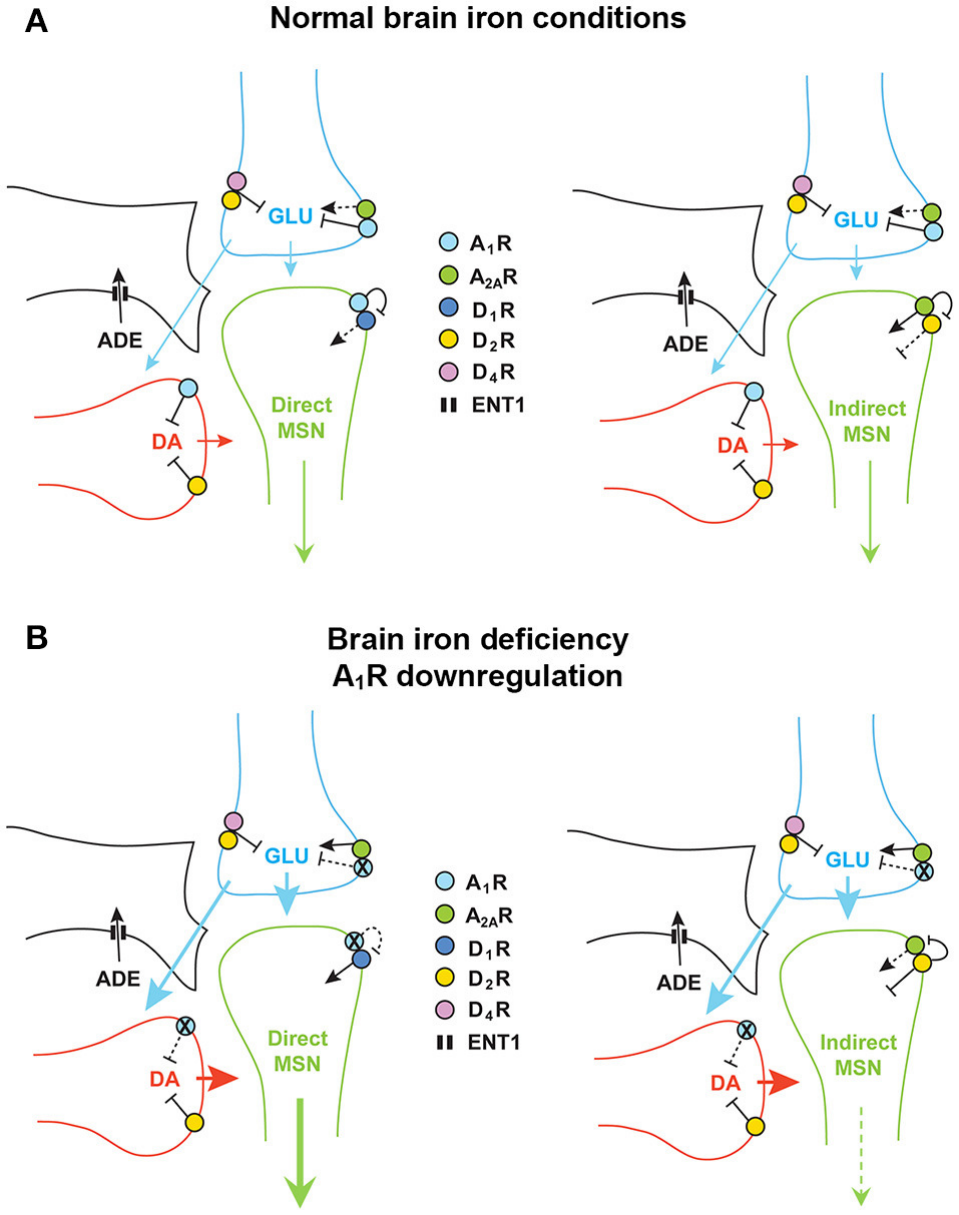
Adenosine and dopamine receptors in the striatal glutamatergic and dopaminergic terminals and in the dendritic spines of the direct and indirect medium spiny neurons (MSN). **(A)** With normal brain iron conditions, extracellular concentrations of adenosine keep an inhibitory presynaptic tone of adenosine on glutamate and dopamine transmission, mediated by A1R, which results in a relatively low activation of the direct and indirect MSN. **(B)** Downregulation of A1R induced by brain iron deficiency leads to hypersensitive glutamatergic terminals, to disinhibition of glutamate and dopamine release, to triatal hyperdopaminergic and hyperglutamatergic states, which leads to an increase and decrease in the activity of the direct MSN and indirect MSN, respectively. DA, dopamine; GLU, glutamate; ADE, adenosine. The equilibrative nucleoside transporter ENT1 (also localized in neurons) is only represented in the astroglial process.

In the brain, the most salient place of interactions of dopamine, glutamate and adenosine is in the striatum, in the striatal GABAergic medium spiny neurons (MSNs), which constitute more than 95% of the striatal neuronal population (Gerfen, 2004). There are two subtypes of MSNs that give rise to the two striatal efferent pathways that connect the striatum with the output structures of the basal ganglia, which are the medial segment of the globus pallidus and the substantia nigra pars reticulata (Gerfen, 2004). The direct MSN constitutes the direct pathway, since directly connects the striatum with the output structures and selectively expresses A1R and D1R, and also D3R in the ventral striatum (Ferré et al., 1996, 1997; Sokoloff and Le Foll, 2017). The indirect MSN connects the striatum with the lateral segment of the globus pallidus and the ventral pallidum, and selectively expresses A2AR and D2R (Ferré et al., 1993, 1997). We have demonstrated that A1R and D1R and A2AR and D2R form specific receptor complexes, the A1R-D1R and A2AR-D2R heteromers (Ferré et al., 1997, 2016; Ginés et al., 2000; Hillion et al., 2002; Canals et al., 2003). The biochemical properties of these heteromers will be analyzed with more detail below, but we can introduce the concept that they act as molecular devices by which endogenous adenosine, by acting on the respective adenosine receptor, tonically inhibits the affinity and signaling of the respective dopamine receptor (see below and Figure 1A).

Apart from the postsynaptic striatal A1R-D1R and A2AR-D2R heteromers, adenosine and dopamine receptors are also localized in the terminals of the main striatal afferents, the dopaminergic and the glutamatergic terminals (Bamford et al., 2004; Borycz et al., 2007; González et al., 2012) (Figure 1A). In the glutamatergic terminals, A1R form heteromers with A2AR and D2R form heteromers with D4 receptors (Ciruela et al., 2006; González et al., 2012; Bonaventura et al., 2017) (Figure 1A). The A1R-A2AR heteromer acts as a concentration-dependent switch, since adenosine has more affinity for A1R than A2AR receptors, which activation inhibits and stimulates glutamate release, respectively. Under basal conditions, adenosine tonically activates predominantly A1R, which inhibits glutamate release. A2AR is activated with higher concentrations of adenosine, which would normally occur upon strong glutamatergic input (which is associated to neuronal and glial co-release of ATP and its conversion to adenosine by ectonucleotidases; Cunha, 2016). Activation of A2AR negatively modulates A1R signaling in the heteromers and promotes the opposite, glutamate release (Solinas et al., 2002; Borycz et al., 2007). The D2R and D4R localized in the glutamatergic terminals and possibly forming heteromers also play a significant role in the tonic inhibitory modulation of striatal glutamate release by dopamine (González et al., 2012; Bonaventura et al., 2017) (Figure 1A). A1R and D2R are also found in dopaminergic terminals without forming heteromers, where they also exert a tonic inhibitory modulation of dopamine release. Glutamate also modulates local dopamine release, as we have recently demonstrated with optogenetic-microdialysis experiments (Quiroz et al., 2016b), by a mechanism that seems to be mostly indirect, involving activation of cholinergic interneurons and activation of nicotinic receptors localized in the dopaminergic terminals (in preparation). Finally, we should consider another player, the astrocytic process, which is involved in the indirect production of extracellular adenosine, by releasing ATP, and that clears up adenosine from the extracellular space by nucleoside transporters, particularly ENT1 (Pascual et al., 2005; Parkinson et al., 2011; Dulla and Masino, 2013; Cunha, 2016) (Figure 1A).

A single alteration in the adenosinergic system, downregulation of A1R, could explain the presynaptic hyperdopaminergic and hyperglutamatergic states in RLS. In particular, downregulation of A1R in the corticostriatal glutamatergic terminals could result in an increased sensitivity of those terminals to release glutamate (Figure 1B). These changes recapitulate those observed in A1R knockout mice that show a significant increase in striatal glutamatergic transmission, due to an increased sensitivity of glutamatergic terminals (Salmi et al., 2005). The increased sensitivity of corticostriatal terminals would facilitate stimulated glutamate release and also, secondarily, dopamine release, which could also be potentiated by downregulation of A1R in the dopaminergic terminals (Figure 1B). In the direct pathway MSN, this should result in stronger neuronal activation, also dependent on downregulation of A1R and disinhibition of D1R previously forming heteromers with the A1R (Figure 1B). We have also previously demonstrated that presynaptic A1R activity facilitates postsynaptic A2AR signaling by keeping a low tone of extracellular dopamine release. Thus, co-administration of A1R and A2AR agonists leads to a significant increase in the activity of the indirect MSN, as measured by *c-fos* and *preproenkephalin* expression (Karcz-Kubicha et al., 2003, 2006). Therefore, in the indirect MSN, presynaptic A1R downregulation should lead to decreased neuronal activity. The increased dopamine release should then lead to a reciprocal interaction in the A2AR-D2R heteromer, by which D2R activation blocks A2AR-mediated signaling through adenylyl cyclase (see below and Figure 1B). Since increased activation of the direct and indirect MSN leads to increase and decrease in motor activity, respectively (Gerfen and Surmeier, 2011), the concomitant respective increase and decrease in the activation of the direct and indirect MSN, could explain the akatisia-like symptoms of PLMS. *In conclusion, one single alteration, A1R downregulation-mediated increased sensitivity of corticostriatal terminals, could produce presynaptic striatal hyperglutamatergic and hyperdopaminergic states, which could be a sufficient pathophysiological mechanism* to explain PLMS in RLS. Downregulation of presynaptic D2R localized in glutamatergic and dopaminergic terminals could also be a significant contributing factor.

Consequently, we hypothesized that BID in rodents produces an increased sensitivity of corticostriatal terminals to release glutamate. In that case, corticostriatal terminals could be a main target for the therapeutic effect of drugs clinically successful in RLS. We tested our hypothesis by using the recently introduced optogenetic-microdialysis method, which involves the use of a modified microdialysis probe with an embedded optogenetic fiber. This method allows the delivery of light surrounding the dialysis membrane, around the same discrete area being sampled for extracellular concentrations of glutamate. In addition, the device allows local perfusion by reverse dialysis of drugs (for details, see Quiroz et al., 2016b). In the first optogenetic-microdialysis study, we injected in the rat prefrontal cortex an adeno-associated virus (AAV) encoding channel-rhodopsin 2 (ChR2) fused to the yellow fluorescent protein (YFP), which allows tracking its localization. After several weeks, ChR2 was expressed by the corticostriatal terminals in the ventral striatum and the implanted optogenetic-microdialysis probe allowed measuring glutamate release by those terminals upon light-induced depolarization. We could then demonstrate that blockade of presynaptic A2AR by perfusion with the A2AR antagonist MSX-3 counteracts optogenetically-induced glutamate release (Quiroz et al., 2016b). For the experiments with BID, we aimed at a more motor-involved striatal area, the dorsal striatal area that receives innervation from the agranular motor cortex. This corticostriatal projection has been anatomically well-defined from different studies analyzing striatal neuronal activation upon cortical-electrical stimulation (Sgambato et al., 1998; Gerfen et al., 2002; Quiroz et al., 2006). A significant glutamate release could be obtained in both iron-deprived animals and controls when using a frequency of stimulation of 100 Hz (Yepes et al., 2017), found to be optimal in previous studies of cortical-electrical and striatal optogenetic stimulation (Gerfen et al., 2002; Quiroz et al., 2006, 2016b). But decreasing the frequency of stimulation to 60 Hz did not produce a significant glutamate release in control animals, although a significant glutamate release could still be observed in the rats with BID (Yepes et al., 2017). These results therefore confirmed the hypothesis of a higher sensitivity of corticostriatal terminals to depolarization-induced glutamate release in the rodent brain with BID. As in our previous study, blockade of A2AR with perfusion with MSX-3 counteracted glutamate release, both in controls at 100 Hz and in iron-deprived animals at 60 Hz (Yepes et al., 2017).

If hypersensitive corticostriatal terminals represent a main pathogenetic mechanism of RLS, they could represent a main target for the therapeutic effect of drugs currently used in RLS. As initial treatment for persistent RLS, the Mayo Clinic Recommendations include either non-ergotic dopamine agonists, such as pramipexole and ropinirole, or α_2_δ ligands, such as gabapentin; for refractory RLS, the recommendations are combination therapy (dopamine agonist + α_2_δ ligands), replenishment of iron stores or considering opioid treatment (empirically found efficient for PLMS) (Garcia-Borreguero et al., 2013; Silber et al., 2013). In fact, perfusion of either the α_2_δ ligand gabapentin or the dopamine agonists pramipexole or ropinirole blocked glutamate release induced by optogenetic stimulation, both in controls (at 100 Hz) and in iron-deprived animals (at 60 Hz) (Yepes et al., 2017). To our knowledge, this represents the first example of a convergence of the two different mechanisms of action of dopaminergic and glutamatergic compounds in the BID rodent. Subsequently, we questioned the identity of dopamine receptor subtypes involved in the pharmacological effect of pramipexole and ropinirole. We have recently reported results of the optogenetic-microdialysis technique in knock-in mice expressing the long intracellular domain of D4.7, the product of a polymorphic variant of the D4R gene (*DRD4*) associated with attention deficit hyperactivity disorder (ADHD) and substance use disorders (SUD) (Bonaventura et al., 2017). When compared with the wild-type mouse D4R, the expanded intracellular domain of the humanized D4R conferred a gain of function, blunting optogenetically-induced corticostriatal glutamate release (Bonaventura et al., 2017). These results confirmed a key role of striatal D4R localized in glutamatergic terminals in the control of corticostriatal glutamatergic transmission. Since previous studies also indicated that D2SR (the short isoform of D2R) is also localized in striatal glutamatergic terminals, probably forming heteromers with D4R (González et al., 2012), we analyzed the effect of different dopamine receptor antagonists on the effect of pramipexole. Co-perfusion with selective D4R or D2R, but not D3R antagonists, counteracted the effect of pramipexole and the optogenetic stimulation could still increase glutamate release both in controls (at 100 Hz) and in iron-deprived animals (at 60 Hz), therefore indicating that D4R and D2R, but not D3R are the main targets of the inhibitory effects of dopamine receptor agonists on striatal glutamate release (Yepes et al., 2017).

## Striatal adenosine receptor heteromers: the A2AR-D2R heterotetramer

It is becoming generally accepted that GPCR receptors form pre-coupled functional complexes that include other receptors with the formation of receptor oligomers. The current definition of receptor oligomer is that of “*a macromolecular complex composed of at least two (functional) receptor units (protomers) with biochemical properties that are demonstrably different from those of its individual components”* (Ferré et al., 2009). To understand these unique biochemical properties, we need to understand the basis of allosterism, which is currently defined as “*the process by which the interaction of a chemical or protein at one location on a protein or macromolecular complex (the allosteric site) influences the binding or function of the same or another chemical or protein at a topographically distinct site”* (Smith and Milligan, 2010). An orthosteric agonist, which binds to the same receptor site than the endogenous transmitter, has two main and independent properties: affinity (the avidity with which it binds to the receptor) and intrinsic efficacy (the power with which the agonist produces its functional response). In classical GPCR allosterism, the allosteric ligand binds to a non-orthosteric site and modifies either of the properties of the orthosteric agonist. In this frame, the GPCR has been usually considered as a monomeric entity. However, accumulating convincing evidence indicates that a main GPCR functional unit is constituted by one GPCR homodimer and its cognate G protein (Ferré et al., 2014).

Allosterism in the frame of GPCR homodimers implies the possibility of *allosteric interactions between orthosteric ligands*, either the same or different ligands. The ligand binding to the first protomer modifies either the affinity or intrinsic efficacy of the second protomer. Cooperativity usually refers to the situation where the ligand binding to the first protomer, decreases the affinity of the same ligand binding to the second protomer. But modulator and modulated ligands can also be different orthosteric ligands, agonists or antagonists (Casadó et al., 2009; Ferré et al., 2014). With GPCR heteromers, with two different protomers, we also have two possibilities: first, the same ligand, when the protomers are two different receptor subtypes, such as dopamine D1R and D3R or D2R and D4R, or adenosine A1R and A2AR; second, two obligatory different ligands, when the two protomers bind different endogenous ligands, such as A2AR and D2R or A1R and D1R (Ferré et al., 2014). One of the first clear clues of this type of allosterism in a GPCR heteromer was obtained from radioligand experiments in membrane preparations from rat striatum, where adenosine A2AR ligands were found to modulate the affinity of D2R ligands in rat striatal membrane preparations (Ferré et al., 1991d). In these experiments, the selective A2AR agonist CGS21680 displaced significantly to the right the competitive inhibition curve of dopamine vs. the D2R antagonist tritiated raclopride, indicating a decrease in the affinity of dopamine for the D2R. This experiment also demonstrated, as simultaneously confirmed by Schiffmann et al. (1991) from *in situ* hybridization experiments, that A2AR and D2R are highly co-localized in the same striatal neuron, the indirect MSN.

The possibility of real allosteric interactions between A2AR and D2R ligands strongly suggested direct intermolecular interactions between both receptors. This was later demonstrated first in artificial systems, in mammalian cells transfected with receptors fused to biosensors that only interact when in very close proximity. In Bioluminescence Resonance Energy Transfer (BRET), there is a transfer of energy from a bioluminescent donor, *Renilla* luciferase (Rluc), to a fluorescent acceptor, such as YFP, and this can only occur when both biosensors are closer than 10 nM. In Bimolecular Fluorescence Complementation (BiFC), two complementary halves of the fluorescent sensor separately fused to the putative interacting receptors should complement and reconstitute YFP and, therefore, its ability to produce fluorescence (Canals et al., 2003; Navarro et al., 2010; Bonaventura et al., 2015). These techniques can then be used to determine the biochemical properties of the GPCR heteromer and, indirectly, to allow their identification in the native tissue. *Our rationale is to identify and disrupt the heteromerization interface, the domains of the receptors that establish intermolecular interactions*. We have in fact found evidence for discrete but strong interactions between intracellular domains and extensive but very specific interactions between transmembrane domains (TMs). From *in vitro* experiments of peptide interactions with mass spectrometry we found evidence for a very discrete but powerful electrostatic interaction between an arginine-rich domain of the third intracellular loop of the D2R and a phosphate group from a specific serine within the tail of the A2AR (Woods and Ferré, 2005). Transfection of receptors with mutations of either of these residues led to a very significant reduction of BRET (Ciruela et al., 2004; Navarro et al., 2010). In addition, the same mutations led to the loss of the ability of the A2AR agonist CGS21680 to decrease the affinity of the D2R agonist quinpirole, which demonstrated that this is, in fact, an allosteric interaction within the A2AR-D2R heteromer (Bonaventura et al., 2015).

*Synthetic peptides with the amino acid sequence of the interacting domains are becoming a very successful tool to selectively disrupt the intermolecular interactions in GPCR heteromers*, and not only *in vitro*, in transfected mammalian cells, but also *in situ*, in native tissues, and *in vivo*, in the experimental animal. For instance, in patch-clamp experiments with slices of the ventral striatum of mice selectively expressing green fluorescence protein (GFP) in D2R-expressing neurons (which allowed the identification of the indirect MSN), CGS21680 acted as a D2R antagonist and blocked the decrease in the neuronal excitability induced by the D2R agonist norpropylapomorphine (NPA; Azdad et al., 2009). This D2R antagonist-like effect of CGS 21680 was then completely counteracted by the intracellular application of a small peptide with a sequence of the epitope containing the interacting phosphorylated serine of the tail of the A2AR (Azdad et al., 2009). The efficacy of CGS21680 to counteract the effect of a high concentration of NPA indicated that the A2AR agonist modulates not only the affinity, but also the efficacy of the D2R agonist (Azdad et al., 2009). These results demonstrate the very significant role of the allosteric interaction within the A2AR-D2R heteromer in the modulation of the function of the indirect MSN. More recently we used peptides with amino acid sequences of TMs to explore the involvement of the transmembrane intermolecular interactions. First, we studied which TM-peptides can disrupt A2AR-D2R heteromerization *in vitro* by BiFC experiments (which were selected over BRET experiments due to the significant interference of Rluc function by the TM-peptides; Guitart et al., 2014). BiFC was selectively disrupted by peptides with the sequence of TM 5 of A2AR and D2R, but not with the corresponding TM7 peptides (Bonaventura et al., 2015). Then, the same specific disrupting peptides were used in experiments with proximity ligation assay (PLA), an antibody-based technique which allows identification of receptor complexes in native tissues (Trifilieff et al., 2011). Notably, the number of complexes was significantly reduced by the specific disrupting peptides, demonstrating the existence of A2AR-D2R heteromers *in situ*, in the striatum (Bonaventura et al., 2015).

The allosteric interaction in the A2AR-D2R heteromer that determines the ability of A2AR agonists to act D2R antagonists and counteract D2R-mediated decrease in the excitability of the indirect MSN could explain many results of previous behavioral studies, such as the ability of adenosine agonists to reduce the locomotor activity induced by D2R agonists in reserpinized mice or the opposite effect with adenosine receptor antagonists, such as caffeine (Ferré et al., 1991a,b). The effect of adenosine antagonists indicated that endogenous adenosine exerts a tonic influence on D2R signaling through the A2AR-D2R heteromer. It is now becoming accepted that the psychostimulant effects of caffeine (a non-selective A1R and A2AR antagonist) and selective A2AR antagonists depend on the blockade of the tonic effect of endogenous adenosine mediated by the A2AR-D2R heteromer (Ferré, 2016). Not surprisingly, we also found that A2AR agonists produce catalepsy, which was counteracted by adenosine antagonists, like theophylline (Ferré et al., 1991c). But it was also counteracted by D2R agonists, indicating the existence of a possible *reciprocal interaction*, by which D2R activation decreases the effect of A2AR activation (Ferré et al., 1991c). In fact, A2AR and D2R are respectively coupled to Gs/olf (Gs for short) and Gi/o proteins (Gi for short) and we would expect an antagonistic interaction at adenylyl cyclase level, the canonical interaction by which activation of a Gi-coupled receptor counteracts adenylyl cyclase activation, cAMP accumulation, induced by activation of a Gs-coupled receptor (Gilman, 1987). We explored this possibility in mammalian cells transfected with A2AR and D2R and found that this is the case, that the D2R agonist quinpirole completely antagonizes CGS21680-induced cAMP accumulation and the concomitant downstream signaling, such as an increase in the expression of the immediate-early gene *c-fos* (Kull et al., 1999). This reciprocal D2R-A2AR interaction could also explain the ability of non-selective adenosine receptor antagonists like caffeine and theophylline, as well as selective A2AR antagonists, to counteract the behavioral effects of D2R antagonists, such as catalepsy induced by haloperidol (Casas et al., 1988; Kanda et al., 1994; Shiozaki et al., 1999; Morelli and Wardas, 2001). This would also imply the existence of *a tone of endogenous dopamine mediated by D2R that counteracts the effects of a tone of endogenous adenosine mediated by A2AR*. The blockade of D2R releases A2AR signaling and it is then endogenous adenosine that produces catalepsy upon haloperidol administration. This has been demonstrated in several *ex vivo* studies, where the increase in striatal expression of *c-fos* induced by a D2R antagonist is blocked by non-selective adenosine receptor antagonists and by selective A2AR, but not A1R antagonists (see for instance Pardo et al., 2013).

**Figure 2:**
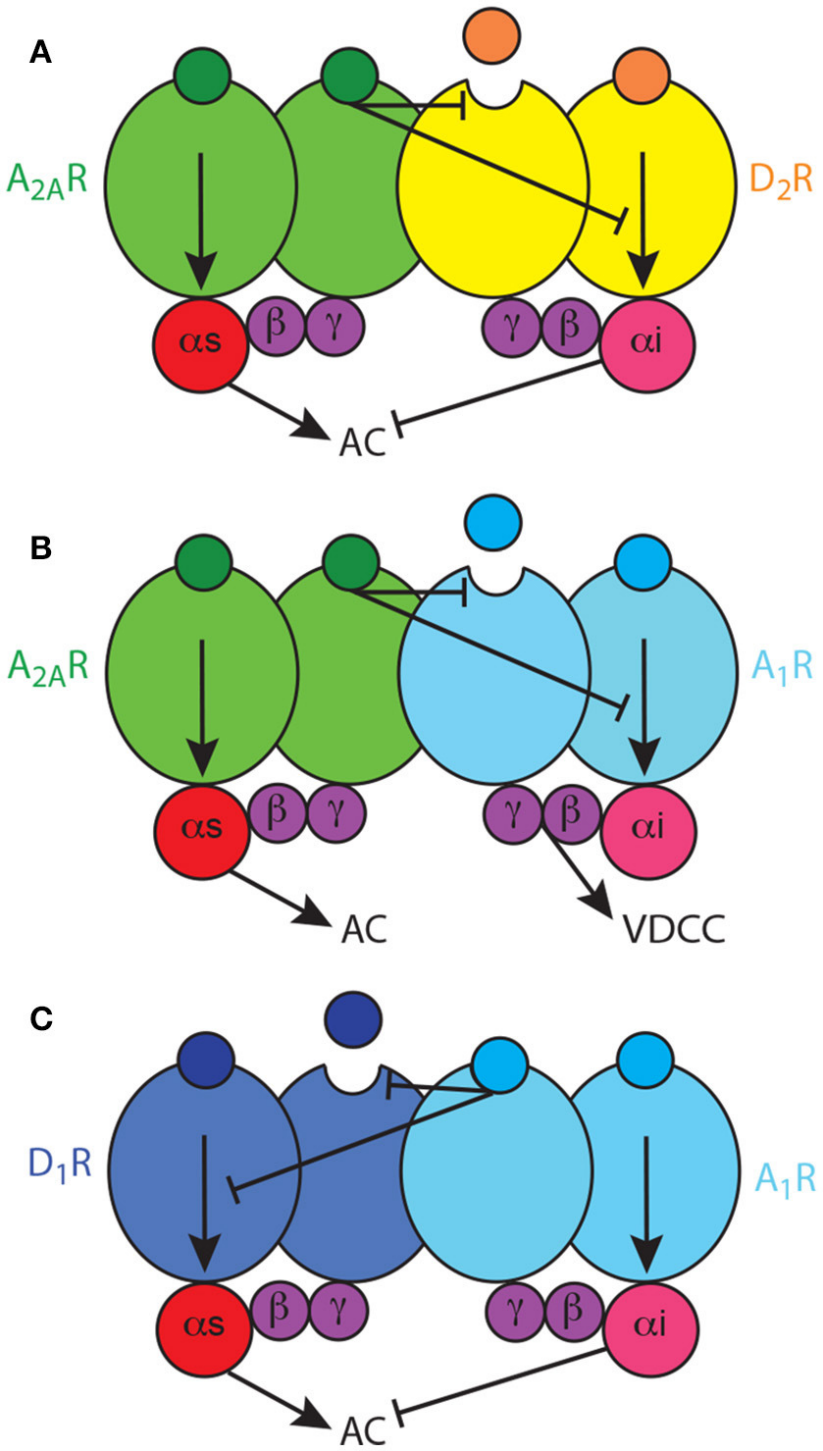
Adenosine Gs-Gi-coupled heterotetramers. **(A)** The A2AR-D2R heterotetramer, constituted by homodimers of the Gs-coupled A2AR and the Gi-coupled D2R, enables two types of reciprocal antagonistic interactions: an allosteric interaction, by which A2AR ligands modulate the affinity and intrinsic efficacy of D2R ligands, and a canonical interaction at the adenylyl cyclase (AC) level, by which D2R agonists inhibit A2AR agonists-mediated AC activation. **(B)** The A1R-A2AR heterotetramer, constituted by homodimers of the Gs-coupled A2AR and the Gi-coupled A1R, enables an allosteric interaction, by which A2AR ligands modulate the affinity and intrinsic efficacy of A1R ligands, but does not enable a canonical interaction at the AC level; A1R signals independently through voltage-dependent Ca^2+^ channels. **(C)** The A1R-D1R heterotetramer, constituted by homodimers of the Gs-coupled D1R and the Gi-coupled A1R, enables unidirectional nonreciprocal allosteric and canonical antagonistic interactions, with A1R ligands modifying the ligand binding properties and adenylyl cyclase activation induced by D1R agonists.

Next obvious question is how two apparently incompatible interactions, the allosteric and the canonical interactions, coexist, and if the canonical interaction also depends on heteromerization. That took us to reconsider the quaternary structure of the A2AR-D2R heteromer, since, just because of steric hindrance, a heterodimer cannot bind simultaneously two G proteins. *We therefore established the following hypotheses: first, that heteromers are often heterotetramers, heteromers of homodimers coupled to their preferred G protein; second, that the heterotetramer enables the canonical antagonistic Gs-Gi interaction; third, that the heterotetramer gives the frame for the pre-coupling of the two receptors involved in the canonical interaction, their respective G proteins and the effector adenylyl cyclase* (Ferré, 2015) (Figure 2A). Using a double complementation assay, with both BiFC and Rluc complementation, fusing the complementary halves of the BRET biosensors to different molecules of A2AR and D2R, we could demonstrate that such a quaternary structure is in fact possible in transfected cells (Bonaventura et al., 2015). Subsequently, we have used all possible 14 TM-peptides corresponding to the seven TMs of A2AR and the seven TMs of D2R and studied the interface of not only A2AR and D2R, but also the A2AR and D2R homodimers in BiFC experiments. Significantly, only one peptide, TM6 of A2A, disrupted A2AR dimerization, and only one peptide, TM6 of D2R, disrupted D2R dimerization. Furthermore, again TM5, but also TM4, of both A2AR and D2R disrupted A2AR-D2R heteromerization (Navarro et al., submitted). Importantly, taking into account these results, as well as the crystal structure of the A2AR, the D3R (as homologous to the D2R) and the β_2_ adrenergic receptor in complex with Gs, computerized modeling allowed only one solution, a linear quaternary structure of the A2AR-D2R heterotetramer, with two internal protomers that provide the heteromeric interface and two external protomers that couple to the alpha subunits of their respective G protein (Navarro et al., submitted).

We have then addressed the possible dependence on heteromerization of the A2AR-D2R canonical interaction in striatal cells in culture, where we previously showed that quinpirole counteracts cAMP accumulation induced by CGS21680 (Navarro et al., 2014). First, we could demonstrate the existence of the A2AR-D2R heteromers in the striatal cultures with PLA. Thus, TM4 and TM5, but not TM6 or TM7, of both A2AR and D2R disrupted the A2AR-D2R complexes (Navarro et al., submitted). Then, only the peptides that disrupted heteromerization disrupted the canonical interaction. Therefore, we could confirm that the canonical interaction is a biochemical property of the A2AR-D2R heterotetramer. *In conclusion, both the allosteric and the canonical interactions are biochemical properties of the A2AR-D2R heterotetramer, which acts as a molecular device that integrates the adenosinergic and dopaminergic signals in the indirect MSN*. The output is mostly determined by the dopaminergic input (high and low for positive and negative reward prediction errors; Ferré, 2017) and amplified by the wining A2AR-D2R interaction, either allosteric or canonical (Ferré, 2016, 2017). In addition, from experiments with BRET and BiFC, fusing the biosensors to the A2AR or D2R and to adenylcyl cyclase type 5 (AC5), we obtained evidence for the pre-coupling of the A2AR-D2R heterotetramer, Gs and Gi proteins and the effector adenylyl cyclase subtype 5 (AC5). Taking into account our results with TM peptides corresponding to the putative TMs of adenylyl cyclase, computer modeling (now also including Gs in complex with the catalytic domains of adenylyl cyclase) suggested that one heterotetramer can bind two molecules of AC and that one molecule of AC can bind two heterotetramers, allowing the formation of high-order oligomers with alternative links of heterotetramers and AC (Navarro et al., submitted).

## Striatal adenosine receptor heteromers: the A1R-A2AR and A1R-D1R heterotetramers

*The A2AR-D2R heterotetramer is the most studied and best characterized GPCR heteromer. Therefore, it can then be used as a model for establishing similarities and differences in the biochemical properties of other GPCR heteromers*. Apart from the A2AR-D2R heteromer, the same tetrameric quaternary structure has been observed for four additional striatal Gs-Gi-coupled heteromers which could also be involved in the pathophysiology of RLS: the A1R-A2AR heterotetramer (Ciruela et al., 2006; Navarro et al., 2016) and the A2AR-CB1R heterotetramer (in preparation), both localized in the striatal glutamatergic terminals, and the D1R-D3R heterotetramers (Fiorentini et al., 2008; Marcellino et al., 2008; Guitart et al., 2014) and the A1R-D1R heterotetramer (in preparation), both localized in the direct MSN. The D2SR-D4R heteromer, on the other hand, is only coupled to Gi proteins and, so far, we do not know if its predominant quaternary structure is dimeric or tetrameric. As mentioned above, the A2AR-D2R heteromer is probably indirectly involved in the dysregulation of striatal function that leads to PLMS symptoms, related to a predominant canonical interaction, the D2R-mediated inhibition of A2AR-mediated signaling, associated to the presynaptic hyperdopaminergic state. But, as also mentioned above, BID-induced downregulation of A1R seems could be a main pathogenetic mechanism in RLS, which should imply a more direct involvement of the A1R-A2AR and A1R-D1R heteromers. Interestingly, the analysis of the structure (homo and heteromeric interfaces) and biochemical properties of the different heterotetramers discloses differences that differ from those of the A2AR-D2R heterotetramer. Significantly, those biochemical differences closely relate to their properties as modulators of neuronal function (excitability, neurotransmitter release).

As mentioned above, the A1R-A2AR heterotetramer acts as a concentration-dependent switch, since adenosine has more affinity for the A1R than for the A2AR, allowing low concentrations of adenosine to inhibit and high concentrations to stimulate striatal glutamate release, by activating A1R and A2AR, respectively (Ciruela et al., 2006). Different from postsynaptic receptors (see above), studies with selective antagonists indicate that striatal presynaptic A2AR are not tonically activated by adenosine while presynaptic A1R are tonically activated, particularly in specific striatal compartments. Thus, A1R antagonists increase, while A2AR antagonists do not modify, the striatal extracellular concentration of glutamate (Solinas et al., 2002; Borycz et al., 2007). This might be related to the higher density of postsynaptic receptors and also indicates that *presynaptic A1R are more sensitive than presynaptic A2AR to the variations of endogenous adenosine*. Clearly, the A1R-A2AR heteromer-mediated concentration-dependent switch mechanism cannot be explained in the frame of a canonical interaction at the level of adenylyl cyclase, where the result of the activation of a Gi-coupled receptor depends on its ability to counteract adenylyl cyclase activation by a Gs-coupled receptor. In fact, we have obtained evidence for the lack of existence of canonical interaction in the A1R-A2AR heterotetramer (Navarro et al., submitted) (Figure 2B). Thus, in the glutamatergic terminals, A1R can signal independently of A2AR within the A1R-A2AR heteromer, most probably by a βγ-dependent-mediated inhibition of presynaptic voltage-dependent Ca^2+^ channels (Wu and Saggau, 1997; Gonçalves and Queiroz, 2008). Yet, when reaching the right concentration to bind A2AR, adenosine inhibits A1R function by a negative allosteric interaction and promotes glutamate release by activating adenylyl cyclase (Ciruela et al., 2006; Gonçalves and Queiroz, 2008) (Figure 2B). The same mechanism has also been described in cortical astrocytes in culture, where A1R-A2AR heteromers modulate GABA uptake (Cristóvão-Ferreira et al., 2013). In addition, evidence for A2AR-D2R heteromers that modulate glutamate release has recently been obtained in striatal astrocytes in culture (Cervetto et al., 2017). The presence and functional significance of these astrocytic adenosine receptor heteromers need still to be determined.

Soon after the discovery of specific pharmacological interactions between A2AR and D2R, similar antagonistic interactions were observed with A1R and D1R ligands, with A1R agonists and antagonists promoting the specific inhibition and facilitation of D1R agonist-mediated locomotor activation, respectively (Ferré et al., 1994, 1996). Similarly, specific biochemical and possibly intermolecular interactions were reported which pointed to the existence of A1R-D1R heteromers (Ferré et al., 1998; Ginés et al., 2000), and which would selectively modulate the function of the direct MSN (Ferré et al., 1994, 1997, 1999). The initial studies in mammalian transfected cells indicated the existence of both allosteric and canonical interactions, but, different to those in the A2AR-D2R heteromer, not reciprocal, with A1R ligands modifying the ligand binding properties and adenylyl cyclase activation induced by D1R agonists (Ferré et al., 1998) (Figure 2C). Using TM peptides, BiFC and PLA experiments, we have now obtained experimental evidence for the tetrameric structure of A1R-D1R, for the dependence on heteromerization for the canonical interaction and for the presence of the heteromer in striatal tissue (Moreno et al. in preparation) and the spinal motoneuron (Rivera-Oliver et al., submitted). The spinal A1R-D1R heterotetamer can explain the recently demonstrated spinally-generated caffeine-induced locomotor activation in rats (Acevedo et al., 2016), and we put forward the hypothesis that it can represent a mechanism involved in the spinal component of RLS (Trenkwalder and Paulus, 2010).

## Targeting adenosine neurotransmission in RLS: the equilibrative transporter ENT1

Apart from the striatum, which could represent a main locus for the alteration of sensory-motor integration in RLS, involved in PLMS symptoms, hypoadenosinergic transmission should also occur in other brain areas. In fact, as mentioned above, we could also demonstrate downregulation of A1R in the cortex of mice with severe and less severe BID (Quiroz et al., 2016a). As mentioned above, adenosine is a main mediator of sleepiness following prolonged wakefulness, which determines its extracellular accumulation in the basal forebrain, cortex, and hypothalamus. Upon activation of A1R, this accumulation leads to inhibition of the cells of origin of the corticopetal basal forebrain system (McCarley, 2007; Ferré, 2010) and the prefrontal corticofugal neurons that innervate the cells of origin of the pontine ascending arousal systems (Van Dort et al., 2009). Upon activation of both A1R and A2AR, adenosine also inhibits the hypothalamic histaminergic and orexinergic ascending arousal systems (McCarley, 2007; Ferré, 2010). BID-mediated A1R downregulation in the basal forebrain, cortex, and hypothalamus, could then be the main pathophysiological mechanism responsible for the hyperarousal and sleep disturbances of RLS. In fact, experimental data strongly suggest that A1R is a marker of the homeostatic sleep response, of the need for recovery of lack of sleep. This includes the rebound sleepiness and the cumulative sleepiness after acute and chronic sleep deprivation, respectively (Bjorness et al., 2009, 2016; Kim et al., 2012). It has in fact been demonstrated that acute and chronic sleep deprivation lead to A1R upregulation in the brain, including both cortex and striatum (Elmenhorst et al., 2007, 2009; Kim et al., 2012, 2015). Finally, as mentioned above, spinal A1R downregulation, and particularly in the motoneuron, could decrease the D1R inhibition, by decreasing the stoichiometry of D1R forming heteromers with D1R.

If A1R downregulation-dependent hypoadenosinergic transmission represents a significant pathogenetic factor in RLS and is directly or indirectly involved in the symptoms of both PLMS and hyperarousal, administration of A1R agonist should represent a successful therapeutic strategy. Unfortunately, A1R agonists cannot be used as direct targets, since they produce very significant peripheral effects, namely pronounced bradycardia and hypotension (Schindler et al., 2005). An alternative strategy would be to increase the adenosine tone below the limit of activation of presynaptic A2AR, with inhibitors of adenosine transporters or adenosine metabolism. Our initial choice is focusing on equilibrative nucleoside transporters. Nucleoside transporters are not only important as a mechanism to salvage extracellular nucleosides for intracellular synthesis of nucleotides, but they are also important as regulators of the extracellular levels of adenosine and as providers of an endogenous tone of adenosinergic neurotransmission mediated by adenosine receptors. In mammals, there are two types of nucleoside transporters, equilibrative and concentrative, which mediate a bidirectional equilibrative transport driven by chemical gradient and a unidirectional concentration transport driven by sodium electrochemical gradient, respectively (Parkinson et al., 2011). Adenosine uptake in the brain occurs primarily by facilitated diffusion via equilibrative transporters, which pharmacological blockade is associated with an accumulation of adenosine in the extracellular space (Parkinson et al., 2011; Dulla and Masino, 2013; Cunha, 2016). From the four types of equilibrative transporters so far identified (ENT1, ENT2, ENT3, and ENT4), ENT1 and ENT2 are the most expressed in the brain, both by neurons and astrocytes (Parkinson et al., 2011). Nevertheless, some studies suggest that ENT1 has a more salient role in determining the concentration of extracellular adenosine in the brain and its dependence on glutamate receptor activation (Alanko et al., 2006; Bicket et al., 2016). Furthermore, of importance for the present discussion, ENT1 (but not ENT2) shows a regional co-localization with A1R, which supports an important role of ENT1-mediated transport of adenosine in the control of the neuromodulatory actions mediated by A1R in the human brain (Jennings et al., 2001).

We can therefore deduce that ENT1 inhibitors could be useful therapeutic agents in RLS. Importantly, some non-selective ENT1/ENT2 inhibitors such as dipyridamole are already being medically used for other clinical purposes. Dipyridamole is used as an inhibitor of platelet aggregation to decrease the risk of thromboembolic complications and recurrence of stroke in patients known to have atherosclerotic cerebrovascular disease. Its effect depends on a combination of mechanisms, including cAMP accumulation in platelets induced by phosphodiesterase inhibition and activation of A2AR by an increased extracellular adenosine secondary to ENT1/2 inhibition in microvascular endothelial cells (Kim and Liao, 2008). However, the possible use of dipyridamole (and other ENT1/ENT2 inhibitors) as a central nervous system agent remains uncertain in view of its reported low ability to cross the blood-brain barrier (Sollevi, 1986; Parkinson et al., 2011) and, to our knowledge, it is not well-established if systemic administration of dipyridamole in the experimental animal leads to behaviorally significant increases in the extracellular levels of adenosine.

**Figure 3:**
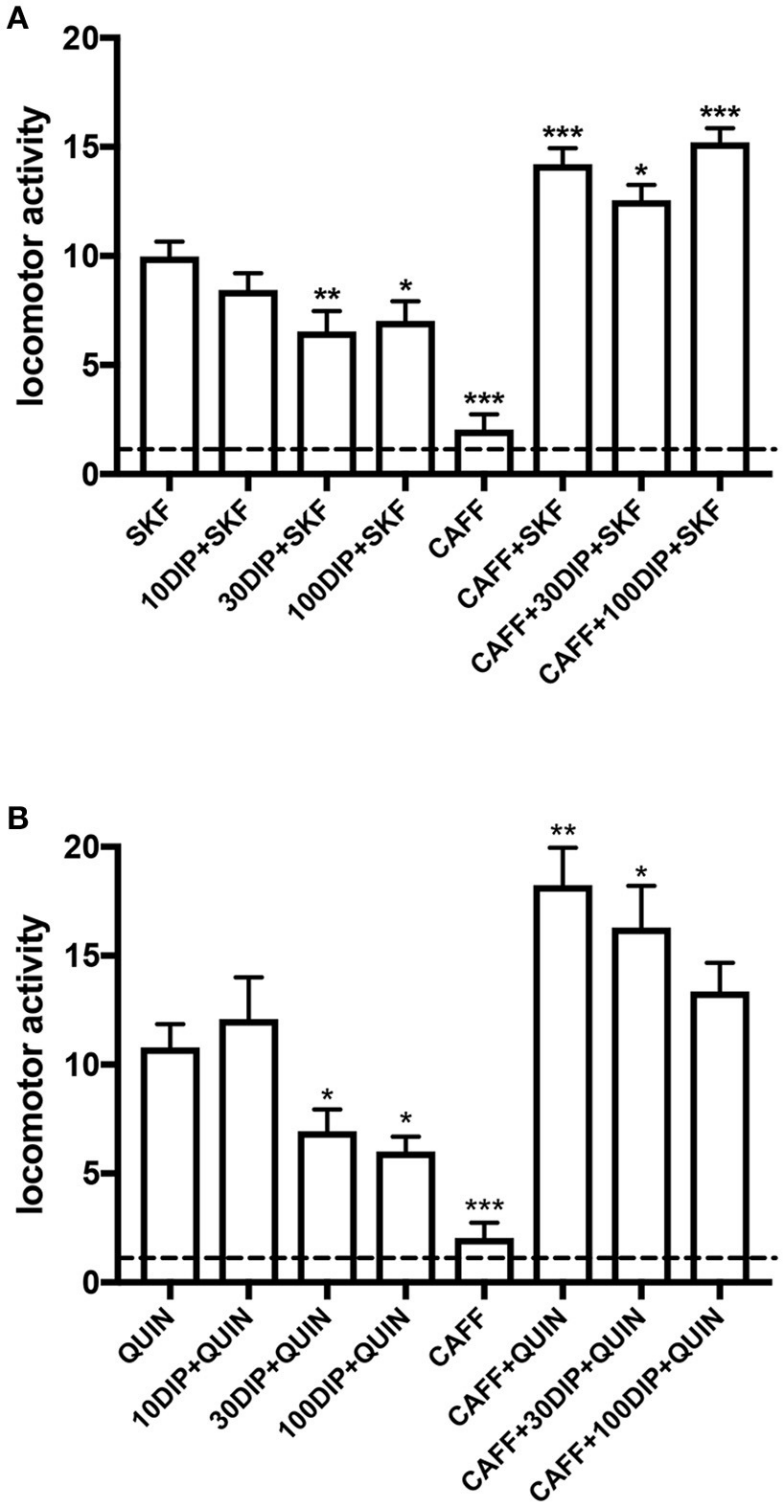
Adenosine-dependent modulation by dipyridamole on the locomotor activation induced by dopamine receptor agonists in reserpinized mice. Locomotor activity in male C57BL/6J mice (20–30 g) 20 h after administration of reserpine (5 mg/kg, s.c.; method described in detail in Marcellino et al., 2008) induced by the D1R agonist SKF81297 (5 mg/kg, i.p.; SKF; **A**) or the D2R agonist quinpirole (5 mg/kg, i.p.; QUIN; **B**), with or without the previous administration of dipyridamole (10, 30 or 100 mg/kg, i.p., 15 min before SKF or QUIN; 10DIP, 30DIP, or 100DIP) or caffeine (30 mg/kg, i.p., 30 min before SKF or QUIN; CAFF). All animals received three i.p. injections, with either drugs or the corresponding vehicle. One reserpinized group also received caffeine without dipyridamole or dopamine agonists. The dashed line represents the average locomotor activity of reserpinized mice receiving only vehicle administrations. Statistical differences were analyzed by one-way ANOVA followed by Newman-Keuls *post-hoc* test; ^*^, ^**^, and ^***^: *p* < 0.05, *p* < 0.01 and *p* < 0.001, respectively, as compared with either SKF or QUIN.

Using the classical reserpinized mice model, we evaluated the ability of the systemic administration of dipyridamole to promote an increase in the adenosinergic tone in the brain. In that case, dipyridamole should counteract the locomotor activating effects of D1R and D2R, by acting on striatal A1R-D1R and A2AR-D2R heteromers (Ferré et al., 1991a,b, 1994), and the effect of dipirydamol should then be counteracted by a non-selective A1R/A2AR antagonist, like caffeine. This model, in fact, has been very useful for the discovery of the specific antagonistic interactions between adenosine and dopamine receptor ligands that led to the discovery the A2AR-D2R and A1R-D1R heteromers. As shown in Figure 3, dipyridamole, at a minimal dose of 30 mg/kg, significantly decreased the locomotor-activating effect of equipotent doses of the D1R agonist SKF81297 and the D2R agonist quinpirole (5 mg/kg in both cases). As expected, caffeine (30 mg/kg) did not produce a significant effect on its own but significantly potentiated the locomotor activation induced by either agonist. In both cases, the depressant effect of dipyridamole was totally counteracted by caffeine (Figure 3). The results, therefore, can entirely be explained by the ability of systemically administered dipyridamole to promote an increase in the basal extracellular levels of striatal adenosine than normally exert a tonic activating effect on postsynaptic A1R-D1R and A2AR-D2R heteromers. Also, such an increase should be expected to increase the activation of presynaptic A1R and hopefully restore the hyperdopaminergic and hyperglutamatergic state in RLS patients. Optogenetic-microdialysis experiments are in progress to demonstrate the ability of dipyridamole to inhibit glutamatergic neurotransmission in hypersensitive corticostriatal terminals in BID rats.

In view of the evidence for the central adenosinergic effect of dipyridamole in reserpinized mice, we explored the possible clinical efficacy of dipyridamole in a prospective 2-month open trial in 13 previously untreated patients diagnosed with idiopathic RLS (García-Borreguero et al., submitted). Therapeutic response was defined as at least a 50% improvement in the “International RLS Scale” and the “Multiple Suggested Immobilization Tests.” Sleep efficiency (SE%), sleep latency and other standard scales were used to evaluate sleep dysfunction and hyperarousal. Dipyridamole was well-tolerated and only two patients had to discontinue at the beginning of the trial due to dizziness. Six and four out of the thirteen patients were full and partial responders, respectively, and only three patients had no significant response. Importantly, not only there was a significant effect of subjective symptoms, but also of PLMS and sleep complaints. These are, of course, preliminary results which could be influenced by a placebo effect and, therefore, await confirmation by a more extensive double-blind clinical trial. Confirmation of the therapeutic effect of dipyridamole in RLS would bring ENT1 inhibition as a new therapeutic approach for RLS, offering an alternative to dopaminergic drugs and, therefore, to their long-term complications, mainly augmentation. This is an overall increase in symptom severity and intensity and represents a common complication of all dopaminergic drugs, with prevalence rates of nearly 50%, and is a common cause of treatment failure (Earley et al., 2014; Ferré et al., 2017). Furthermore, our preliminary results suggest that, in contrast to dopaminergic agonists, ENT1 inhibitors should be effective not only for the treatment of dysesthesias and PLMS, but also for sleep complaints and hyperarousal in RLS. In addition to dipyridamole, there are other marketed compounds with ENT1 inhibitory activity already used at the clinical level for their vascular relaxation and platelet inhibition (ticagrelor, dilazep) or their anti-inflamatory effects (sulindac). The challenge would nevertheless be to obtain new potent and selective ENT1 (or ENT1/ENT2) inhibitors with significant brain penetration.

It conclusion, the main tenet of this essay is that a main mechanism responsible for PLMS and hyperarousal in RLS can be a BID-induced hypoadenosinergic state, with downregulation of A1R. This mechanism may disrupt the adenosine-dopamine-glutamate balance uniquely controlled by adenosine and dopamine receptor heteromers in the striatum and also the A1R-mediated inhibitory control of glutamatergic neurotransmission in the cortex and other non-striatal brain areas and in the spinal cord. We then provide preclinical and clinical evidence for a possible new alternative therapeutic strategy for RLS, increasing the adenosinergic tone in the CNS with ENT1 inhibitors.

## Ethics statement

All animals used in the study were maintained in accordance with the guidelines of the National Institutes of Health Animal Care and the animal research conducted to perform this study was approved by the NIDA IRP Animal Care and Use Committee (protocol #: 15-BNRB-73).

## Author contributions

WR, AS, and DG-B performed the experiments; SF, XG, AS, WR, and DG-B analyzed data; SF, WR, and DG-B designed the experiments; SF, CQ, WR, EM, VC-A, MD-R, VC, SC, RA, CE, and DG-B wrote the manuscript.

### Conflict of interest statement

The authors declare that the research was conducted in the absence of any commercial or financial relationships that could be construed as a potential conflict of interest.
